# Treatment following Triple-AAV Delivery in Mature Murine Model of Human *CDH23*-Associated Hearing Loss

**DOI:** 10.3390/cimb45120590

**Published:** 2023-11-24

**Authors:** Hidekane Yoshimura, Shu Yokota, Yutaka Takumi

**Affiliations:** Department of Otorhinolaryngology—Head and Neck Surgery, Shinshu University School of Medicine, Matsumoto 390-8621, Japan

**Keywords:** gene therapy, genetic hearing loss, adeno-associated virus, progressive hearing loss

## Abstract

This study aimed to investigate the transduction efficiency of triple adeno-associated virus (AAV) vectors in the cochleae of adult mice, focusing on large-gene-associated hearing loss (HL). Additionally, we sought to evaluate the feasibility of cochlear gene therapy in a mouse model of human *CDH23*-mediated HL using the triple AAV approach. To create a reporter protein, we fused EGFP to mCherry, which was then divided into three parts, each packaged in a separate AAV2/2 vector. Four weeks after co-injecting the triple AAV vectors into 4–5-week-old mice, we assessed transduction efficiency. We found that up to 5.9% of inner hair cells were positive for both EGFP and mCherry. Subsequently, we developed triple *Cdh23* AAV vectors for therapeutic purposes. After administering these vectors to 4- to 5-week-old C57/BL6 mice, we conducted auditory tests and immunohistochemistry studies over a period of 60 weeks. Co-injecting triple *Cdh23*-AAVs did not alter auditory function or lead to hair cell degeneration. In conclusion, this study confirms the feasibility of the triple-AAV approach for cochlear gene delivery. While this strategy did not produce any treatment effects, our findings suggest that large deafness genes could be potential future targets for cochlear gene therapy.

## 1. Introduction

Congenital hearing loss (HL) is a relatively common disorder occurring in 1–2 per 1000 newborns with over half of the cases being hereditary [1]. Currently, hearing aids (HAs) and cochlear implants (CIs) serve as effective therapeutic devices for HL; however, they are not biological treatments. Gene therapy holds promise as a curative therapeutic method with the potential to suppress HL progression or restore hearing function, something unattainable with HAs or CIs [2]. Several reports have demonstrated successful outcomes of cochlear gene therapy in restoring auditory function in mouse models of genetic deafness [3,4,5,6,7,8,9]. As a result, a Phase-1/2 clinical trial for gene therapy in individuals with *OTOF*-mediated hearing loss, the first in humans, began in 2023 (https://classic.clinicaltrials.gov/ct2/home accessed on 20 August 2023). *OTOF*-mediated HL exhibits congenital severe-to-profound HL but is associated with neurotransmission rather than cochlear architecture, making it a promising target for assessing therapeutic effectiveness [10]. However, achieving successful gene therapy for patients with profound congenital HL may be extremely challenging, except for the *OTOF* gene. To expand the scope of target genes for cochlear gene therapy, patients with progressive genetic HL are considered suitable candidates in terms of the therapeutic time window for gene therapy intervention [2]. When selecting a gene-therapy strategy, the genetic material must be delivered to the inner ear using a vector. While various viral or non-viral vectors have been reported, adeno-associated virus (AAV) vectors are commonly used in most hereditary HL studies due to their nonpathogenicity and minimal immunogenicity [11]. However, AAV is limited in its transfer capacity, capable of transferring only up to 4.7 kb. Since numerous diseases result from gene variants with coding sequences that exceed this capacity, packaging into a single AAV capsid is currently unfeasible for larger genes, potentially requiring the splitting of the transgene into two or three parts [10]. To address this, a dual-AAV-vector approach has been previously reported [12]. Although other laboratories have reported triple-AAV vector delivery in a mouse retina [13], such studies in cochleae remain scarce and warrant further investigation.

*CDH23* is a common deafness gene that can cause either Usher syndrome type 1D (USH1D) or non-syndromic HL (DFNB12). The phenotype range of DFNB12 is variable from congenital to adult-onset HL [14,15]. Adult-onset, *CDH23*-related HL is progressive, beginning as high-frequency HL that gradually affects low frequencies, ultimately resulting in HL across all frequencies [16]. While this gene is an ideal target for cochlear gene therapy, the size of the *CDH23* coding sequence is 10.1 kb; therefore, the development of gene therapy using triple AAV vectors is necessary [13]. In this study we aimed to investigate the transduction efficiency of triple-AAV vectors in the cochleae of adult mice and evaluate the feasibility of cochlear gene therapy in a mouse model of human *CDH23*-mediated HL using a triple-AAV approach.

## 2. Materials and Methods

### 2.1. Virus Production

**Figure 1:**
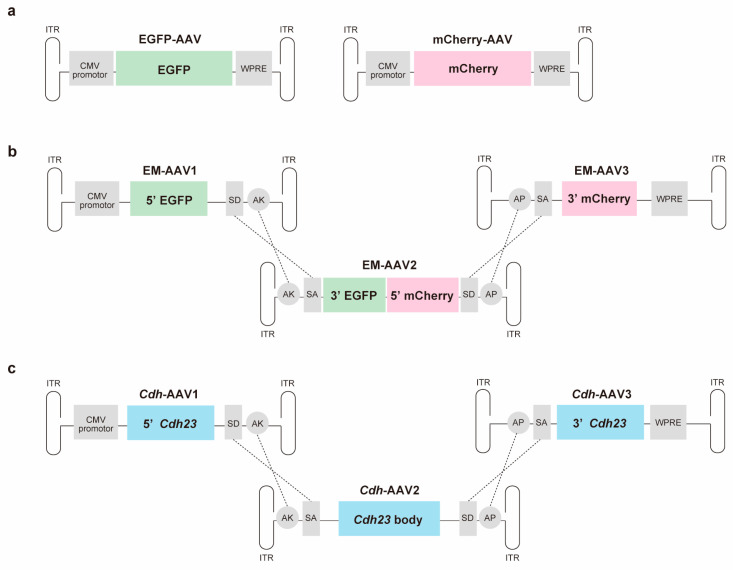
Schematic representation of generated single- and triple-AAV vectors: (**a**) Single AAV; (**b**) Triple EM-AAVs; (**c**) Triple *Cdh*-AAVs. ITR, inverted terminal repeat; SD, splicing donor signal; SA, splicing acceptor signal; AP, alkaline phosphatase recombinogenic region; and AK, F1 phage recombinogenic region.

All AAV vectors were prepared by Vector Builder (Chicago, IL, USA). To validate the injection method, we developed [9] and compare its transduction efficiency with triple-AAV vectors; two types of single-AAV vectors were generated: AAV2/2 with a CMV-driven EGFP or mCherry transgene cassette. The viral titers were AAV2/2.CMV.EGFP (EGFP-AAV) at 1.10 × 10^13^ GC/mL and AAV2/2.CMV.mCherry (mCherry-AAV) at 1.49 × 10^13^ GC/mL (Figure 1a).

For the triple injection study, we created a reporter protein by fusing eGFP to mCherry under the CMV promoter. This protein was then split into three parts, each packaged in a separate AAV2/2 vector, following the method described by Maddalena et al. [13]. In brief, to generate the triple EGFP-mCherry-AAV (EM-AAV) vector, the EM CDS was split into three constructs: (1) EM-AAV1 containing the CMV promoter and the N-terminal EGFP CDS (bp 1–393); (2) EM-AAV2 containing the C-terminal EGFP CDS (bp 394–717) and the N-terminal mCherry CDS (bp 1–307); (3) EM-AAV3 containing the C-terminal DsRed CDS (bp 308–711) plus the WPRE cassette (Figure 1b). The viral titers were AAV2/2.CMV.EM1 (EM-AAV1) at 1.75 × 10^13^ GC/mL, AAV2/2.CMV.EM2 (EM-AAV2) at 1.46 × 10^13^ GC/mL, and AAV2/2.CMV.EM3 (EM-AAV3) at 1.91 × 10^13^ GC/mL. For recombinogenic regions at the 3′ end of AAV-EM1 and the 5′ end of EM-AAV2, we used the AK sequence derived from the phage F1 genome (J02448.1, bp 5850–5926). Additionally, we placed the AP sequence (NM_001632.4, bp 1802–1516) derived from the human placental alkaline phosphatase gene at the 3′ end of EM-AAV2 and the 5′ end of EM-AAV3. The splice donor (SD) and splice acceptor (SA) signals contained in the triple AAV vector are as follows: 50-GTAAGTATCAAGGTTACAAGACAGGTTTAAGGAGACCAATAGAAACTGGGCTTGTCGAGACAGAGAAGACTCTTGCGTTTCT-30 (SD); 50-GATAGGCACCTATTGGTCTTACTGACATCCACTTTGCCTTTCTCTCCACAG-30 (SA).

Similarly, we generated triple-*Cdh23^c.753G^* AAV vectors as therapeutic vectors. Three fragments of *Cdh23^c.753A^* (NM_023370.3) were designed as described by Maddalena et al. (2017). *Cdh*-AAV1 contains the N-terminal CDS (bp 1–3369), *Cdh*-AAV2 contains the body of the CDS (bp 3370–6712), and *Cdh*-AAV3 contains the C-terminal CDS (bp 6713–10,065), followed by the WPRE cassette (Figure 1c). Note that we used the *Cdh23* CDS sequence with c.753G modified by the *Cdh23^c.753A^* CDS (NM_023370.3) because the *Cdh23^c.753G^* allele results in normal exon splicing, while the *Cdh23^c.753A^* allele in C57BL/6J mice disrupts the canonical donor splice site sequence and causes in-frame exon skipping [17]. The viral titers were AAV2/2.CMV.Cdh23_1 (*Cdh*-AAV1) at 1.44 × 10^14^ GC/mL, AAV2/2.CMV.Cdh23_2 (*Cdh*-AAV2) at 5.67 × 10^13^ GC/mL, and AAV2/2.CMV.Cdh23_3 (*Cdh*-AAV3) at 2.88 × 10^13^ GC/mL. Aliquots of the virus were stored at −80 °C and thawed before use.

### 2.2. Animal Models

The mice were housed in a temperature-controlled environment with a 12 h light/dark cycle. C57BL/6J mice were purchased from Japan SLC (Shizuoka, Japan). The C57BL/6J mouse model of progressive HL carries the homozygous splice-site variant c.753A in the *Cdh23* gene, known as the *ahl* allele (*Cdh23^ahl/ahl^*). HL in C57BL/6J (*Cdh23^ahl/ahl^*) mice progresses gradually from the high-frequency range and spreads toward low frequencies, resulting in severe-to-profound HL, indicating that they possess a phenotype similar to that of humans with *CDH23*-related HL [18].

### 2.3. Animal Surgery

C57BL/6J mice (4–5-weeks-old) were used for both single- and triple-AAV injection studies and gene therapy experiments. A *trans*-RWM injection combined with canal fenestration (RWM+CF injection) was performed as previously described [9]. Briefly, the mice were anesthetized using an intraperitoneal injection of ketamine (100 mg/kg) and xylazine (10 mg/kg). A postauricular incision was made to access the temporal bone. After exposing the ventral cochlear bulla to the facial nerve, the posterior semicircular canal (PSCC) was exposed dorsally to the cochlear bulla. A 0.5 mm diameter otologic drill (Minimo One Series Ver.2, Minitor Co., LTD, Tokyo, Japan) was used to drill a small hole in the cochlear bulla, which was then widened sufficiently with forceps to visualize the stapedial artery and the RWM. A hole was drilled in the PSCC using a 0.5 mm diameter diamond drill, and slow egress of the perilymph confirmed a patent canalostomy. After waiting for 5–10 min for perilymph egress to abate, 1.0 μL of AAV vectors with 2.5% fast green dye (Sigma-Aldrich, St. Louis, MO, USA) was loaded into a GD-1 glass capillary (1.0 mm outer diameter [OD] × 0.6 mm inner diameter [ID]; Narishige, Tokyo, Japan) pulled using a PC-100 puller (Narishige) and affixed to a microinjector (Narishige). For the triple-AAV injection study and gene therapy experiments, 0.5 μL of each AAV vector (total: 1.5 μL) was loaded. The pipettes were controlled manually using a micropipette manipulator. The RWM was gently punctured in the center, and AAV was microinjected into the scala tympani for a couple of minutes. Successful injections were confirmed by visualizing the efflux of green fluid from the PSCC canalostomy. After removing the pipette, the RWM niche was quickly sealed using a small muscle plug to prevent leakage. The bony defects of the bulla and canal were sealed using small muscle plugs and Vetbond™ tissue adhesive (3M, Maplewood, MN, USA). A total of 6–0 absorbable polypropylene sutures and 6–0 nylon monofilament sutures were used to close the SCM and skin, respectively. The total surgical duration ranged from 30–40 min. All animals were operated on by a single surgeon (HY). All animal studies were approved by the Institutional Animal Care and Use Committee of Shinshu University School of Medicine (approval no. 020116).

### 2.4. Auditory Testing

ABRs were recorded as previously described [9,12]. All mice were anesthetized using an intraperitoneal injection of ketamine (100 mg/kg) and xylazine (10 mg/kg). All recordings were conducted from both ears of all animals on a heating pad, using electrodes placed subcutaneously in the vertex and underneath the left or right ear. Clicks were delivered as square pulses of 100 ms duration, and tone bursts were 3 ms in length at distinct frequencies of 8, 16, and 32 kHz. ABRs were measured using BioSigRZ (Tucker-Davis Technologies, Alachua, FL, USA) for both clicks and tone bursts, adjusting the stimulus levels in 5-decibel (dB) increments between 25–90 dB sound pressure levels (SPL) in both ears. Electrical signals were averaged over 512 repetitions. The ABR threshold was defined as the lowest sound level at which a reproducible waveform was observed. For transduction efficiency analysis, ABRs were measured 4 weeks after the injection and for gene therapy experiments at 12, 24, 36, 48, and 60 weeks of age. Responses from the contralateral ear that did not undergo surgery were used as controls.

### 2.5. Immunohistochemistry, Cell Counts, and Transduction Efficiency Analysis

All injected and non-injected cochleae were harvested after euthanasia via CO_2_ inhalation. For transduction efficiency analysis, cochleae were harvested 4 weeks after injection and for gene therapy experiments at 60 weeks of age. Temporal bones were locally perfused and fixed in 4% paraformaldehyde overnight at room temperature (25 °C), rinsed in 1× PBS (#14190250, Thermo Fisher Scientific, Rockford, IL, USA), and stored at 4 °C in preparation for immunohistochemistry. The specimens were visualized using a dissection microscope and dissected for whole-mount analysis. Infiltration was performed using 0.3% Triton X-100 for 30 min, followed by blocking with 5% normal goat serum for 1 h. For single- and triple-AAV injection studies, EGFP and mCherry were detected by their intrinsic fluorescence in all cochlear whole mounts. Tissues were incubated with rabbit polyclonal myosin-VIIA antibody against hair cells (#25-6790, Proteus Biosciences Inc., Ramona, CA, USA), diluted 1:200 in PBS, and stored overnight at room temperature. Subsequently, for single- and triple-AAV injection studies, fluorescence-labeled goat anti-rabbit IgG Alexa Fluor 405 (#A-31556, Thermo Fisher Scientific) at a 1:500 dilution was used as the secondary antibody for 1 h at room temperature. For the gene therapy experiments, fluorescence-labeled goat anti-rabbit IgG Alexa Fluor 568 (#A-11036, Thermo Fisher Scientific) at a 1:500 dilution was used as a secondary antibody for 1 h at room temperature. The specimens were mounted in ProLong™ Diamond Antifade Mountant (#P36961, Thermo Fisher Scientific) and observed using a Leica TCS SP8 confocal microscope (Leica Microsystems Inc., Bannockburn, IL, USA). Cell count and transduction efficiency analyses were performed as previously described. Z-stack images of whole cochlear mounts were collected at 10× magnification using a Leica SP8 confocal microscope. Maximum intensity projections of the Z stacks were generated for each field of view, and images were prepared using LAS X (Leica Microsystems Inc.) to meet equal conditions. For single- and triple-AAV injection studies, IHCs with positive EGFP and/or mCherry and overlapping Myo7a were counted per 400 μm cochlear section in each specimen using the ImageJ Cell Counter (NIH Image). The total numbers of HCs and EGFP- and/or mCherry-positive HCs were summed and converted to percentages. For gene therapy experiments, IHCs positive for Myo7a were counted per 400 μm cochlear section for each turn in each specimen using the ImageJ Cell Counter. The total number of HCs was summed and converted into percentages.

### 2.6. Statistical Analysis

Sample sizes are noted in the figure legends. Statistical analyses were performed using the Prism 8 software package (GraphPad, San Diego, CA, USA). Two groups were compared using an unpaired two-tailed Student’s *t*-test. Statistical significance was set at *p* < 0.05.

## 3. Results

### 3.1. Injection of a Single AAV Vector Demonstrates Robust Transgene Expression in IHCs

**Figure 2:**
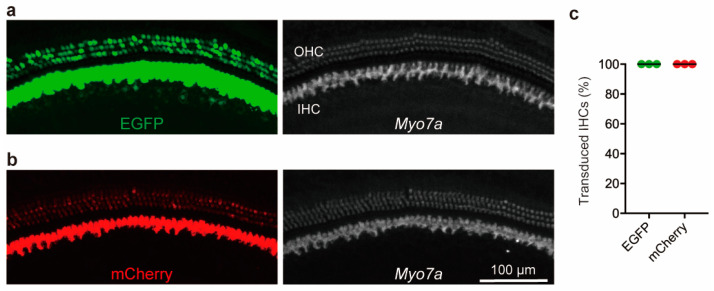
Single-AAV vector delivery via RWM + CF injection leads to robust IHC transduction in murine mature cochlea. Representative images of whole-mount apical turns following delivery of single-AAV vector (either AAV2/2.CMV.EGFP (*n* = 3) (**a**) or AAV2/2.CMV.mCherry (*n* = 3) (**b**)). (**c**) Quantitative data of IHC transduction evaluated using EGFP or mCherry expression assessed in 400 μm segments.

To validate our injection approach in this study, the 1.0 μL injection of each single AAV vector (EGFP-AAV, and mCherry-AAV) in the left ear at 4–5 weeks of age using the RWM+CF approach was performed. Four weeks later, both ears were harvested, and cochlear EGFP or mCherry expression was quantitated in whole-mount preparations. Delivery of EGFP-AAV (1.10 × 10^13^ GC/mL) led to high IHC (100%) and OHC transduction in the cochlea (Figure 2a). Delivery of mCherry-AAV (1.49 × 10^13^ GC/mL) led to high IHC (100%), whereas mCherry expression in OHCs was limited (Figure 2b). These findings suggest that a single AAV delivery using the RWM+CF approach facilitates high and uniform transduction of IHCs in the mature murine cochlea, which is consistent with our earlier report [12].

### 3.2. Injection of Triple AAV Vector Demonstrates Limited IHC Transduction

**Figure 3:**
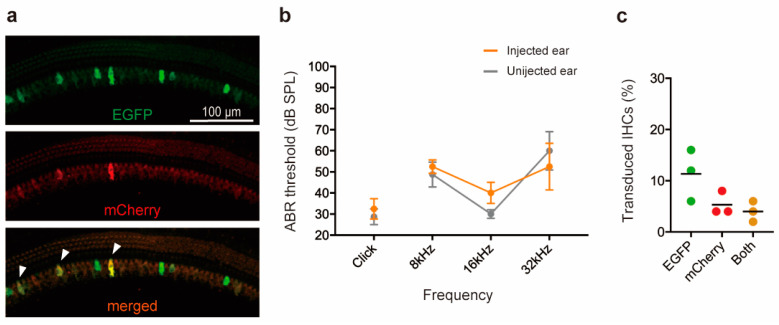
Cochlear gene delivery by triple-AAV vectors allows the IHC transduction in murine mature cochlea. (**a**) Representative images of whole-mount apical turns following delivery of triple EM-AAV vectors using RWM + CF injection. Cochleae were harvested 4 weeks after the inoculation (delivered at 4 to 5 weeks of age), imaged for native EGFP (green) and mCherry (red). Arrowheads indicate transduced IHCs. (**b**) Click and tone-burst ABR thresholds in injected ears (*n* = 3) and uninjected contralateral ears (*n* = 3) 4 weeks after the injection. Data are means ± SEM. (**c**) Quantitative data of IHC transduction evaluated using EGFP, mCherry, or both expressions assessed in 400 μm segments.

Subsequently, to investigate the transduction efficiency using triple AAV vectors, we co-injected 0.5 μL of each AAV vector (EM-AAV1, 2, 3; 7.65 × 10^13^ GC/mL) into the left ear of 4- to 5-week-old mice using the RWM+CF technique. Four weeks later, we harvested both ears and quantified each co-transduction ratio using whole-mount preparations. We found that up to 15.7% of IHCs across the cochlea (with variations in different cochlear regions: apex up to 15.4%, middle up to 12%, and base up to 15.7%) were positive for EGFP alone. This EGFP expression resulted from the concatemerization of EM-AAV1 and 2. Up to 7.8% of IHCs throughout the cochlea (with regional variations: apex up to 1.9%, middle up to 4%, and base up to 7.8%) were positive for mCherry alone. This mCherry expression arose from the concatemerization of EM-AAV2 and 3. Furthermore, up to 5.9% of IHCs throughout the cochlea (with regional variations: apex up to 1.9%, middle up to 2%, and base up to 5.9%) were positive for both EGFP and mCherry. This dual expression resulted from the concatemerization of EM-AAV1, 2, and 3 (Figure 3a). Additionally, we did not observe any OHC transduction following the triple AAV approach. The auditory thresholds in the injected and non-injected ears were comparable (Figure 3b).

### 3.3. Delivery of Triple Cdh23-AAV Vectors Does Not Alter Hearing Deterioration in Cdh23^ahl/ahl^ Mice

**Figure 4:**
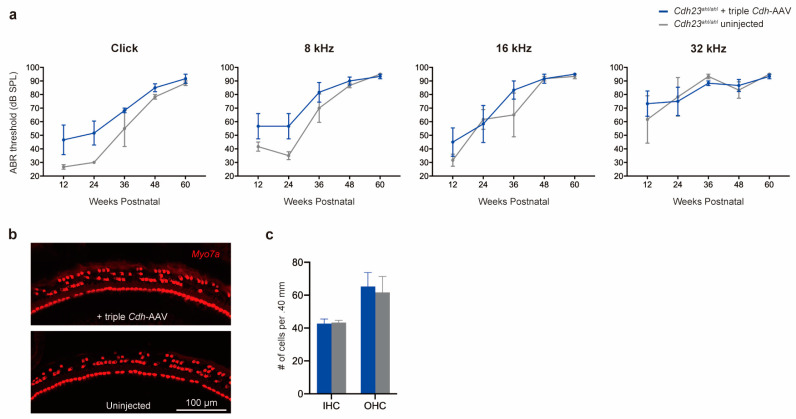
Delivery of triple *Cdh23*-AAV vectors does not impact auditory function and hair cell degeneration in *Cdh23^ahl/ahl^* mice. (**a**) Click and tone-burst ABR thresholds in injected ears (*n* = 3) and uninjected contralateral ears (*n* = 3) 60 weeks after the injection. Data are means ± SEM. (**b**) Representative images of whole-mount apical turns following delivery of triple *Cdh*-AAV vectors by RWM + CF injection. Cochleae were harvested 60 weeks after the inoculation (delivered at 4 to 5 weeks of age) and stained using *Myo7a* (red) to label hair cells. (**c**) Quantitative comparison of IHC survival in injected (blue) and uninjected ears (gray).

After confirming the feasibility of the triple-AAV approach for cochlear gene delivery, we evaluated the effect of administering triple *Cdh23*-AAV vectors in a mouse model of human *CDH23*-related HL. We injected *Cdh*-AAV1, 2, and 3 into the left ear of 4- to 5-week-old *Cdh23^ahl/ahl^* mice using the RWM+CF approach. Auditory function was measured through click- and tone-burst-evoked ABRs in two groups of mice (*Cdh23^ahl/ahl^* uninjected and *Cdh23^ahl/ahl^*+triple *Cdh*-AAV vector). At 60 weeks of age, hearing deterioration was observed in both treated and untreated ears. We found no statistically significant differences in click and tone-burst ABRs between the groups; however, in treated ears, the ABR thresholds of click, 8 kHz, and 16 kHz were elevated compared to those in untreated ears (Figure 4a). Additionally, as the *Cdh23* gene expresses HC in the cochlea, histological analysis was conducted at 60 weeks of age. Hair cells were labeled using an anti-Myo7a antibody, and their count in 400 µm sections allowed us to quantify the effect of triple *Cdh23*-AAV vectors on hair cell survival in two groups. The results indicated that IHC and OHC survival rates were comparable between injected and non-injected ears (Figure 4b).

## 4. Discussion

Approximately 120 deafness genes responsible for non-syndromic HL have been identified (https://hereditaryhearingloss.org/, accessed on 20 August 2023). Gene therapy using AAV vectors is the most promising therapeutic strategy for genetic HL. However, to prevent exceeding the packaging capacity of AAV [4], several deafness genes must be divided. To address this challenge, Akil et al. [14] demonstrated that the dual AAV approach led to high IHC transduction in mature mice with *Otof*-related HL (*OTOF* CDS, 6.0 kb). The efficacy of the dual-AAV vector approach, with a maximum transfer capacity of approximately 9 kb, opens the possibility of further expansion to triple-AAV vectors, theoretically capable of transferring approximately 14 kb. In ophthalmology, Maddalena et al. [13] showed that the triple AAV approach, using subretinal administration, was effective. While no triple AAV vector approach to the inner ear has been reported to date, we present the first successful application of this strategy in a mature murine inner ear. However, the transduction rate in IHC was limited, similar to that in mouse photoreceptors [13], suggesting that the triple-AAV approach resulted in a much lower IHC transduction level compared to a single AAV injection. Furthermore, our triple-AAV approach did not uniformly succeed, which aligns with a previous study on the dual-AAV approach [12]. The reasons for these differences in transduction efficiencies remain unclear.

To translate animal research into clinical applications for humans, attention must be given to the differences in inner-ear development between mice and humans. In humans, the inner ear matures at 26 weeks of gestation, while in mice it does not mature until 15 days after birth. Therefore, it is challenging to directly apply the results of studies using neonatal mice to humans, necessitating experiments with adult mice [9,19]. However, only two papers, targeting *Otof* [3] and *Tmc1* [9], demonstrated positive outcomes. To expand the number of target genes for cochlear gene therapy, we selected *CDH23* for this study. Currently, for patients with *CDH23*-related HL, CIs are highly effective but not biologically based treatments. Moreover, because most patients with *CDH23*-related HL exhibit severe-to-profound high-frequency HL with only mild-to-moderate HL at low frequencies, electric acoustic stimulation (EAS) was developed to provide acoustic stimulation amplifying low-frequency residual hearing while delivering electrical stimulation through a CI to improve high-frequency hearing loss with a single device [20]. In such patients, preserving residual hearing at low frequencies is crucial for enhancing speech recognition in noise, music appreciation, and sound localization [21,22]. Consequently, even if gene therapy for *CDH23*-related HL can suppress HL progression, it remains clinically feasible. To address this, we aimed to treat a mouse model of human *CDH23*-related HL, known as C57/BL6 mice (*Cdh23^ahl/ahl^* mice), using a triple AAV approach. Unfortunately, when compared with untreated ears, the auditory thresholds in the treated ears were statistically similar or slightly elevated. These results suggest that the co-injection of multiple AAV vectors generated truncating proteins, potentially affecting both the safety and functionality of the treatment. Although an auditory threshold shift four weeks after triple EM-AAV was not observed, it is necessary to evaluate the potential toxicity of truncating products due to the triple AAV approach, as previously described [13]. Additionally, the number of surviving IHC in the treated and untreated ears was statistically comparable, indicating that the therapy had no impact.

A potential explanation for our results could be poor transduction in IHC that played a key role in relaying sound information to the auditory nerve using the triple-AAV approach. Additionally, poor transduction in OHC might not lead to the rescue of progressive HL. We believe that there are potential strategies to improve the efficiency of cochlear gene therapy for large deafness genes: (1) Modifying the design and serotype of AAVs for the triple-AAV approach; (2) Exploring minigenes to generate shortened versions of the protein of interest, enabling a switch from a triple- to a single- or dual-AAV approach [7]; (3) Utilizing gene editing techniques like the CRISPR/Cas9 system, which can employ dual-AAV vectors for gRNA and Cas9 protein but requires the design of specific gRNAs for each targeted variant; (4) Using helper-dependent adenoviral (HdAd) vectors, which have a large packaging capacity (~36 kb) [23]. Despite the potential of HdAd vectors, their efficient transduction of the organ of Corti, where deafness gene expression was not observed, indicates the need for improvement in the capabilities of HdAd. Additionally, we recognized that successful delivery and expression of the administrated *Cdh23* gene to the cochlea should be evaluated; however, it was very difficult to distinguish endogenous *Cdh23* (c.753A) from delivered *Cdh23* (c.753G) via immunohistochemistry because only one SNP was different between both genotypes. RNA-seq is one potential strategy which can address this issue. We, therefore, plan to analyze this in our future studies.

## 5. Conclusions

This study is the first to report cochlear gene delivery in the mature murine inner ear using triple-AAV vectors; however, treatment using this strategy has shown no significant effect. While the levels of transduction via triple-AAV vectors are limited, our findings suggest that, by employing the triple-AAV approach, additional deafness genes could potentially serve as future targets for cochlear gene therapy.

## Data Availability

Additional data are available from the corresponding author upon request.
